# Krill oil: nutraceutical potential in skin health and disease

**DOI:** 10.3389/fnut.2024.1388155

**Published:** 2024-07-12

**Authors:** Lan Duo, Jianzhong Yang, Xue Wang, Gang Zhang, Jiuxiang Zhao, Hong Zou, Zhi Wang, Yu Li

**Affiliations:** ^1^CAS Engineering Laboratory for Nutrition, Shanghai Institute of Nutrition and Health, University of Chinese Academy of Sciences, Chinese Academy of Sciences, Shanghai, China; ^2^Jiangsu Sunline Deep Sea Fishery Co., Ltd, Lianyungang, Jiangsu, China; ^3^Shanghai Engineering Research Center of Molecular Therapeutics and New Drug Development, School of Chemistry and Molecular Engineering, East China Normal University, Shanghai, China

**Keywords:** krill oil, skin health, anti-aging, anti-inflammatory, wound healing, antioxidant, ultraviolet protection

## Abstract

Krill oil (KO), extracted from the Antarctic marine crustacean *Euphausia superba*, is a nutrient-dense substance that includes rich profiles of *n*-3 polyunsaturated fatty acids (*n*-3 PUFAs), phospholipids (PLs), astaxanthin (ASX), as well as vitamins A and E, minerals, and flavonoids. As a high-quality lipid resource, KO has been widely used as a dietary supplement for its health-protective properties in recent years. KO has various benefits, including antioxidative, anti-inflammatory, metabolic regulatory, neuroprotective, and gut microbiome modulatory effects. Especially, the antioxidant and anti-inflammatory effects make KO have potential in skin care applications. With increasing demands for natural skin anti-aging solutions, KO has emerged as a valuable nutraceutical in dermatology, showing potential for mitigating the effects of skin aging and enhancing overall skin health and vitality. This review provides an overview of existing studies on the beneficial impact of KO on the skin, exploring its functional roles and underlying mechanisms through which it contributes to dermatological health and disease management.

## Introduction

Antarctic krill (*E. superba*) is a small, marine crustacean from the Antarctic Ocean. It is one of the most abundant organisms in the world, with a total biomass of up to 1 billion tons. Krill is the cornerstone of Antarctic ecology, serving as food for most fish. More importantly, despite its small size, with adults reaching only lengths of 4.5–6 cm, krill is remarkably valuable. Its unique nutritional composition has attracted intense research interest, not only in metabolic regulation (1), cardiovascular health (2), brain development (3) but also in skin health (4).

The skin is the largest organ of the human body, which consists of three layers: epidermis, dermis, and subcutaneous. It protects against ultraviolet (UV) rays, physical trauma, and external microbial environment and prevents moisture loss (5). Problems with skin health are related to many factors, including individual phenotype, lifestyle choices (e.g., alcohol consumption and lack of sleep), environmental factors (e.g., UV rays and pollutants), and various diseases (6). Skin aging is categorized into two types: intrinsic aging, which refers to the natural cellular aging process leading to wrinkles and pigmentation linked to oxidative stress, and external aging, predominantly resulting from environmental factors, most notably UV-radiation-induced photoaging (7, 8). The cutaneous immune system exerts a protective effect by responding to inflammatory stimuli (9). Exposure to multiple environmental factors, such as UV rays and microorganisms, activates the skin's inflammatory response (10). The cutaneous immune system is regulated by mediators such as cytokines and bioactive lipids. However, when immune responses are inadequate or mounted against non-infectious agents, it leads to chronic inflammation, which is a feature of many pathological skin conditions (11). Skin trauma includes a range of damages, including wounds, burns, scars, etc., which are caused by various factors such as physical, chemical, microbial, and pathological (e.g., diabetic foot). The skin condition during the wound-healing process is critical for chronic wound management. Thus, discovering new therapeutic approaches for skin problems remains a significant challenge (12).

Skin health is dependent on adequate nutrition. Vitamins, minerals, and essential fatty acids are thought to improve skin problems caused by both internal and external causes (13). With increasing demands for solutions to skin problems, research focusing on natural ingredients that enhance skin beauty and health is advancing the fields of dermatology and nutrition. Compared with chemically synthesized cosmetics, natural active substances extracted from natural plants, herbs, marine organisms, etc., have the advantages of being more natural, pure, and hypoallergenic. In particular, they have strong beneficial effects on the skin, such as UV radiation protection, antioxidant activity (14–16), regulating epidermal protection barrier (17–19) and maintaining water–ion balance (20, 21). Natural active substances, including aloin (22, 23), ginsenosides (24, 25), hydroxycinnamic acids (26, 27), and astaxanthin (ASX) (28, 29) have been proven to effectively solve a series of skin problems. Increasing research demonstrated that oral administration of some active substances also enhances skin performance, while traditional beauty and skin care products are generally used topically. Collagen is orally taken to improve skin elasticity and reduce wrinkles (30). Dietary supplements rich in antioxidants and anti-inflammatory ingredients are expanding their use for skin health. Using phospholipids (PLs) as a carrier can better penetrate cell membranes, promote human body absorption, and increase bioavailability (31, 32). Therefore, it is necessary to explore more natural substances with efficacious, safe, natural, and sustainable features, and develop novel products for skincare to meet market concerns and demands (33).

Krill oil (KO), extracted from Antarctic krill, is showing its potential in skin care applications due to its unique characteristics. KO is a nutrient-dense substance, including *n*-3 polyunsaturated fatty acids (*n*-3 PUFAs), PLs, ASX, as well as vitamins A and E, minerals, and flavonoids (34). In the past decade, KO has been approved as a safe food by the US Food and Drug Administration (FDA), European EFSA, and China, and has been approved for safe use in pregnant and lactating women. KO has been widely used as a dietary supplement for its health-protective properties in recent years. Especially the active ingredients of *n*-3 PUFA and ASX have anti-inflammatory and antioxidant properties, and PLs, as an important component of the cell membrane, can enhance the bioavailability of other effective ingredients. These three components have been widely used in the field of skin health (35–37). Recently, research on cellular, animal, and clinical trials of the benefits of KO on skin health has gradually emerged. This paper reviews for the first time the effects and potential mechanisms of KO and its ingredients on skin health, including anti-aging, anti-inflammation, and wound-promoting, which aim to provide new directions for skin nutrition research and skin health and disease management.

## Ingredients in krill oil that benefit skin health

**Figure 1:**
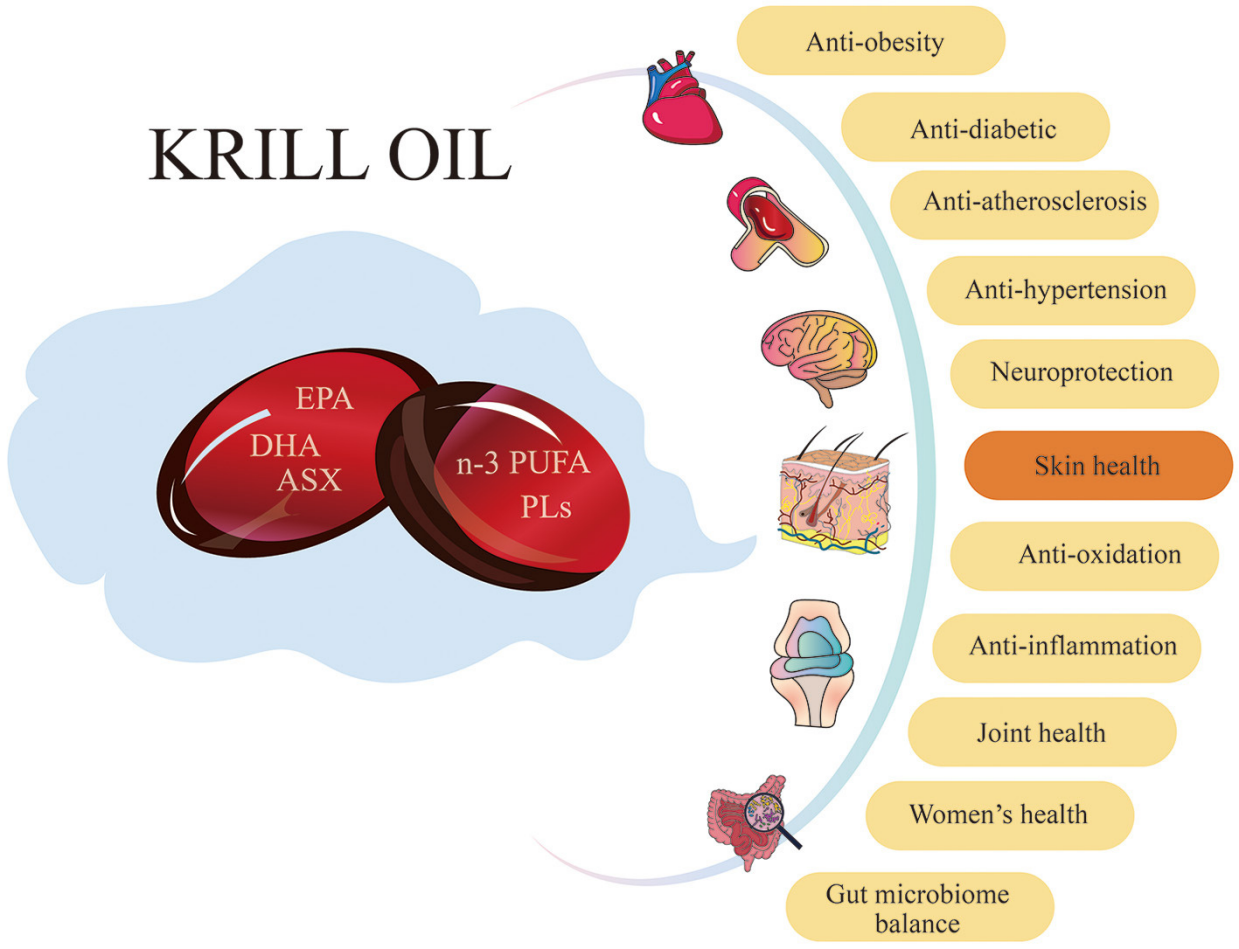
Krill oil has a variety of benefits for human health. KO has positive effects on numerous aspects of human health, including antioxidant, anti-inflammation, anti-obesity, anti-diabetic, anti-atherosclerosis, and anti-hypertension. Additionally, it provides neuroprotection, improves skin health and joint health, benefits women's health, and maintains gut microbiome balance.

KO has been widely used as a nutritional supplement to improve human health in recent years. Increasing research shows that KO has various benefits, including antioxidant (4, 38, 39), anti-inflammatory (40–42), metabolic regulation (1, 43, 44), neuroprotective (3, 45, 46), and gut microbiota balance (47–49) (Figure 1). Especially, the antioxidant and anti-inflammatory effects make KO have potential in skin care applications.

Compared with other oils from marine organisms, KO has a unique composition and proportion (Table 1). Lipids account for 0.4%−3.6% of the content of fresh krill (34). Depending on the sample type and analysis method, the phospholipid content of the extracted KO ranges from 39.29% to 80.69% (50), with 30%−65% fatty acids incorporated into PLs (54). Eicosapentaenoic acid (EPA) (ranging from 14.3% to 28%) and docosahexaenoic acid (DHA) (ranging from 7.1% to 15.7%) are the most abundant fatty acids found in KO (34). ASX content is about 40–5000 mg/kg (50, 52, 55).

**Table 1:** Concentration of main ingredients in krill oil (KO).

**KO ingredients**	**Concentration**	**References**
Omega-3 polyunsaturated fatty acids (*n*-3 PUFA)	21.5–56.57%	(34, 50)
Eicosapentaenoic acid (EPA)	14.3–28%	(34, 50)
Docosahexaenoic acid (DHA)	7.1–15.7%	(34, 50)
Phospholipids (PLs)	39.29–80.69%	(34, 51)
Phosphatidylcholine (PC)	44.58–99.80%	(34)
Phosphatidylethanolamine (PE)	0.20–24.74%	(34)
Lysophosphatidylcholine (LPC)	0.51–44.39%	(34)
Astaxanthin (ASX)	40 to 5000 mg/kg	(50, 52, 53)

The efficient extraction method of KO is still being actively explored. Some studies have reported that the lipid yield increased to 4.18% after two-step chitinase/protease hydrolysis. Each gram of KO contains 246.05 mg of phospholipids, 80.96 mg of free EPA and DHA, and 0.82 mg of ASX (56). Unsaturated fatty acids in KO are very easy to oxidize (57, 58). Further research on the extraction process and storage stability of KO will help improve the content, proportion, and activity of the active ingredients in KO.

### Omega-3 polyunsaturated fatty acids (*n*-3 PUFA)

DHA and EPA are the most important *n*-3 PUFA in KO. DHA is a 22-carbon fatty acid with six double bonds. EPA refers to a 20-carbon fatty acid with five double bonds. KO contains both PLs and triglycerides (TAG), and the content of PLs is as high as 65% (59). In KO, the majority of EPA (31.13%) and DHA (14.87%) bind to PLs, while a small portion of DHA (1.5%) and EPA (3.17%) bind to TAG (60). Since the cell membrane is constructed of PLs, DHA is usually esterified to phosphatidylcholine (PC) or phosphatidylethanolamine (PE) at the sn-2 position, which significantly increases the absorption of DHA and EPA in KO (52, 61, 62). The ratio of DHA and EPA in KO is about 1:2 (59). It has been found in cellular, animal, and clinical trials that EPA can resist skin aging, be anti-inflammatory, and promote wound healing, not only used as a dietary supplement but also topically used on the skin (63–65), which inferred that the effect of KO on improving skin health may be related to the high ratio of EPA. Compared with fish oil (FO), KO has higher efficiency, showing similar benefits with smaller doses. FO is primarily composed of TAG, DHA, and EPA; and FO mainly binds to TAG; and TAG-bound DHA/EPA cannot accumulate in membrane phospholipids. In addition, the ratio of DHA and EPA is about 1:1 in FO, with similar total fatty acid content to KO (52, 59).

Research has shown that *n*-3 PUFA can affect the neurological development of fetuses and infants (66), improve retina (67), provide cardiovascular protection (68), enhance memory function (69), improve anxiety, and reduce inflammatory markers (70). Increasing research has shown that *n*-3 PUFA, primarily EPA and DHA, are known to affect the structure and function of the skin (71). It can provide photoprotection and reduce UV-induced skin problems, including skin hydration, erythema, occlusion, and inflammatory problems, and even reduce the risk of skin cancer (72). *n*-3 PUFAs, to a certain extent, have many aspects of anti-inflammation, including leukocyte chemotaxis, production of inflammatory cytokines, and lymphocyte reactivity (73). Clinical trials have confirmed that dietary supplementation of PUFA can significantly improve inflammatory skin diseases such as acne (74) and psoriasis (75). DHA and EPA intake is associated with an 80% reduction in the risk of melanoma (76). DHA is superior to the effect of EPA in promoting wound healing (77). In addition, studies have proven that EPA can not only be used as a dietary supplement but also as a topical application, which expands the application scope and effectiveness of PUFA. EPA can increase the efficacy of cannabidiol (CBD). After 56 days of EPA and CBD application on subjects' faces, the area and number of erythema were significantly reduced, and skin hydration and elasticity increased by 31.2% and 25.6%, respectively (78). This may be related to the ability of PUFA to improve aging by oxidizing the telomere-reverse transcriptional gene axis (79). Topical application of EPA has a significant impact on UV-induced skin damage and intrinsic aging (80).

### Astaxanthin (ASX)

ASX (3,30-dihydroxy-b, b-carotene-4,40-dione) is a type of carotenoid and a red pigment that is responsible for the color of KO. Because the structure of ASX contains both hydroxyl and ketone groups, it shows extremely high resistance to peroxyl radicals (81). ASX has strong antioxidant activity, ten times more than other carotenoids (82). The exact content is mainly related to the krill raw materials, extraction process, and analysis technology. The content of ASX in KO will decrease with increasing storage time. Changing the storage environment conditions or microencapsulation treatment of KO might delay this phenomenon (83, 84).

ASX was proven to prevent cardiovascular diseases (85), protect the nervous system (86), strengthen the immune system to improve aging (87), and prevent cataracts (88). Also, ASX has been extensively studied to improve skin health and combat skin aging, including photoprotection, antioxidation, and anti-inflammation. The production of reactive oxygen species (ROS) is one of the key processes of skin aging (28). ASX pretreatment can reduce the ROS induced by UVB. ASX can also effectively inhibit skin cancer and reduce skin dehydration and wrinkles (89). It is worth noting that animal experiments have shown that ASX could reach the skin layers, including the dermis and epidermis, after oral consumption of ASX (35). At present, beauty products using ASX as raw materials have occupied a certain market and are sought after, including capsules or oral liquids for internal use and skin care products for external use. Several clinical studies have shown that ASX improves skin condition and delays skin aging by exerting its antioxidant and anti-inflammatory effects, suppressing hyperpigmentation and photoaging, reducing transepidermal water loss (TEWL), increasing skin elasticity, and reducing skin wrinkles (42, 90–92). The research showed that ASX has stronger antioxidant and antipollution effects than other natural crocin and other synthetic antioxidants such as alpha-tocopherol (AT), butylhydroxytoluene, butylhydroxyanisole and gallic acid, and is more effective in protecting human skin (93).

### Phospholipids (PLs)

Phospholipids (PLs) are an important component of cell membranes (94). KO contains a large amount of PLs, and its content can be as high as 80.69%, depending on the sample type and analysis method. The most abundant PLs in KO include phosphatidylcholine (PC) (44.58% to 99.80%), phosphatidylethanolamine (PE) (0.20% to 24.74%), and lysophosphatidylcholine (LPC) (0.51% to 44.39%) (Table 1) (34).

As mentioned above, increasing evidence showed that PLs might be a more effective delivery form of *n*-3 PUFAs to body tissues than TAGs (95, 96). The presence of PLs in KO greatly improved the bioavailability of KO and the absorption of fatty acids, especially DHA and EPA (52). It is confirmed that EPA and DHA in PL form have better biological activity than triacylglycerol (TAG) or ethyl ester (EE) forms, and can play a role in brain function, antitumor activity, lipid metabolism, and glucose metabolism (97). Lysophosphatidic acid (LPA) is the simplest phospholipid found in nature, not only it can adjust skin function, hair follicle development, wound healing, and skin itching, but also it can play an important role in scleroderma and skin cancer (94).

PLs are commonly used as carrier substances in the field of skin health. PLs can enhance solubility, increase skin permeability, and assist in enhancing photoprotection (31, 32, 98). Luteolin is a natural antioxidant that can absorb a wide range of UV rays, but its water solubility and skin permeability are insufficient. Adding a certain proportion of phospholipids can improve the solubility, permeability, and photoprotective activity of luteolin (98). Vismodegib (VSD) is approved for the prevention and treatment of skin cancer, but its bioavailability is poor. The VSD gel containing phospholipids, ethanol, and terpenes enhanced the skin permeability of VSD, which resulted in a 3.59-fold increase in bioavailability compared to oral VSD, and exhibited excellent antitumor effect (32). Phospholipids and anti-skin cancer substances have synergistic effects to a certain extent, enhancing their bioavailability and anti-skin cancer effect (31).

In summary, the *n*-3 PUFA, ASX, and PL present in KO each contribute distinctive benefits to skin health. Nonetheless, when combined within KO, they work synergistically to produce superior effects that surpass the benefits of any single component alone.

## Krill oil on skin anti-aging

**Figure 2:**
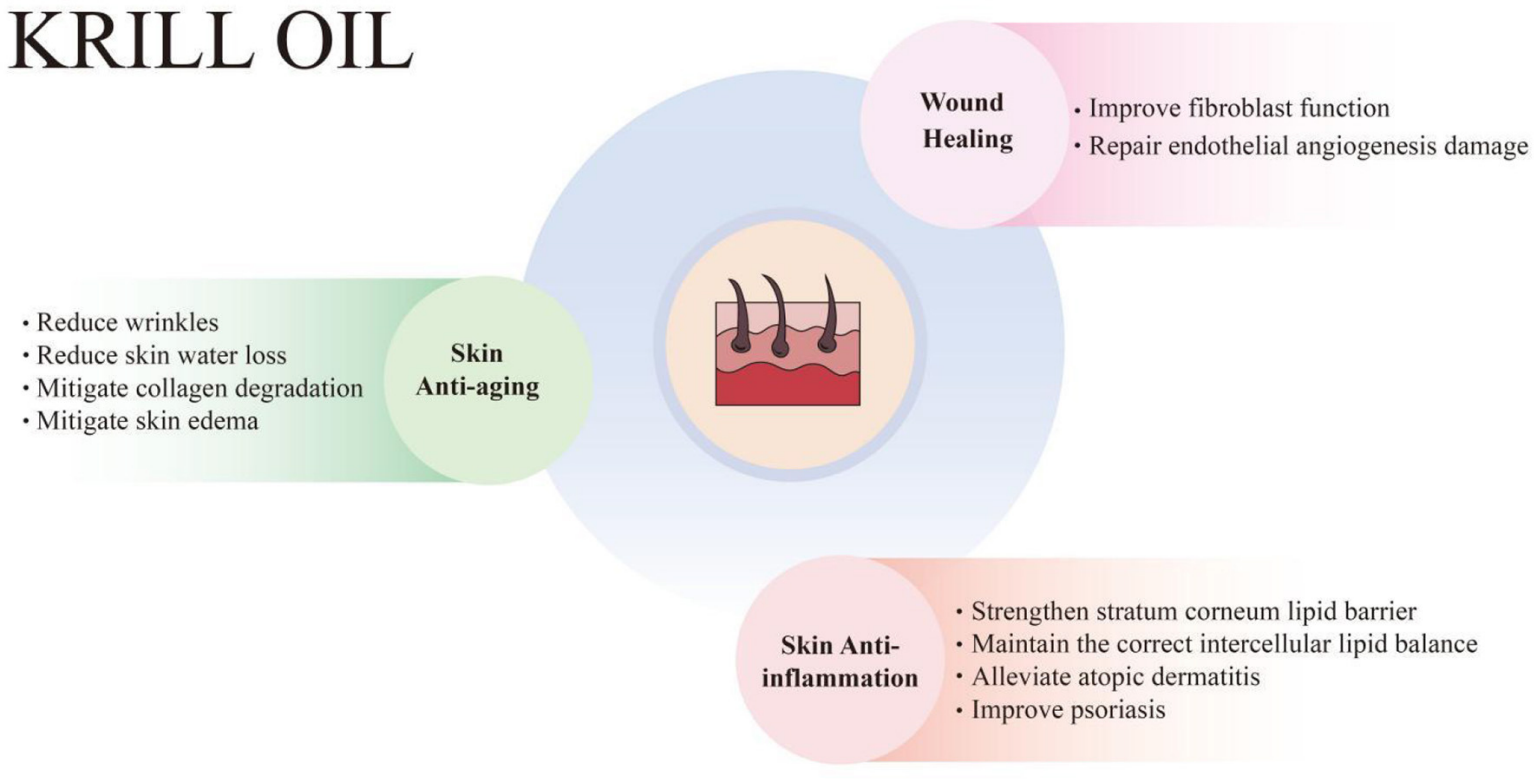
Krill oil has protective effects on skin health, including skin anti-aging, anti-inflammation, and wound healing enhancement. KO combats skin aging, manifesting in the reduction of wrinkles, skin water loss, collagen degradation, and skin edema. KO also relieves inflammatory skin diseases, including atopic dermatitis and psoriasis, by strengthening the stratum corneum lipid barrier and maintaining the correct intercellular lipid balance. Additionally, KO promotes wound healing by improving fibroblast function and repairing endothelial angiogenesis damage.

Skin aging is an inevitable process, which means the skin function gradually weakens and the regeneration ability of skin cells gradually disappears. It manifests as wrinkles, reduced skin moisture, reduced skin elasticity and pigmentation (99, 100). Skin aging can be categorized as chronological aging and photoaging (or internal aging and external aging), and is affected by intrinsic factors and extrinsic factors. Intrinsic factors refer to the aging of skin cells, which are mainly related to time, genetics, hormones, etc. Extrinsic factors include UV exposure, air pollution, nutritional factors, etc. (101). Photoaging, caused primarily by long-term exposure to UV radiation from sunlight, is clinically characterized by deep wrinkling, dryness, pigmentation, and laxity. UVA (320–400 nm) can penetrate the dermis, resulting in damage to dermal collagen and elastin, while UVB (280–320 nm) mainly affects the epidermis and causes DNA damage. UV radiation can cause cell damage by generating ROS and inflammatory mediators, which directly attack the DNA of skin cells (102). ROS are a major factor of skin aging, involving damage to DNA, the inflammatory response, reduced production of antioxidants, and the generation of matrix metalloproteinases (MMPs) that degrade collagen and elastin in the dermal skin layer, leading to skin wrinkles and reduced elasticity (103). Therefore, dietary supplementation with antioxidants and anti-inflammatory agents, especially those that can accumulate in the dermis, could protect the skin from UV-induced damage. Research has demonstrated that KO effectively combats skin aging, as evidenced by its ability to reduce wrinkles, prevent skin water loss, slow down collagen degradation, and alleviate skin edema (Figure 2).

### Reduce wrinkles

Photoaging results in skin histological changes, collagen fiber injuries, and uneven pigmentation, normally known as wrinkled and coarse skin (102). UV causes an increase in matrix metalloproteinases (MMPs), promotes extracellular matrix (ECM) protein degradation, and forms wrinkles.

Human skin cells exposed to UV irradiation were used to study photoaging *in vitro*. KO was evaluated for potential effect on wrinkle improvement and antioxidant activity in human dermal fibroblasts (HDF) cells. HDF exposed to UVB irradiation at 50 mJ/cm^2^ for 2 min induced a significant increase in MMP-1 activity. However, HDF cells treated with KO (0.5–8 mg/mL) at the same time resulted in a significant decrease in MMP-1 activity. Additionally, KO showed a concentration-dependent (from 0.25 to 8 mg/ml) increase in elastase inhibitory activity and procollagen synthesis in HDF. Moreover, KO was found to significantly increase DPPH radical scavenging activity (4). KO showed the protective effects on wrinkle formation in photoaging mice induced by UV radiation. Hairless female mice exposed to UVB radiation thrice a week for 15 weeks induce photoaging symptoms. However, after oral administration of KO (100, 200, or 400 mg/kg) every day for 105 days, the skin wrinkles were significantly inhibited, with the length and depth of wrinkles alleviated. Moreover, skin collagen I (COL1) contents were significantly increased, and the gene expression of MMP-1, MMP-9, and MMP13 in UV-radiated skin tissue was significantly decreased by KO treatments (4).

Wang et al. performed whole-genome proteomics and lipidomics analyses using a photoaging mouse model with *n*-3 PUFA or *n*-6 PUFA supplementation. Significant alleviation of photoaging was observed. They found that *n*-3 PUFA may alleviate photoaging by upregulating Hmmr (hyaluronic acid receptor) expression, which can decrease Mmp9 expression, reducing collagen degradation (36). Clinical studies have confirmed that topical application of EPA can significantly improve the characteristics of photoaging. Kim et al. applied 2% EPA or solvent to the buttock skin of young men for topical application twice, 24 h apart each time, and used 2 MEDs UV irradiation 24 h after the second application (MED, minimum erythema dose: 70–90 mJ/cm^2^). Skin biopsy results showed that EPA significantly improved UV-induced skin epidermal thickening. Immunohistochemistry and western blot analysis showed that UV reduced the procollagen expression of fibroblasts in the upper dermis and dermoepidermal junction. EPA restored the expression of procollagen after UV irradiation. Procollagen levels were similar to control levels. EPA significantly inhibited the expression of MMP-1 and MMP-9 after UV treatment, inhibited c-Jun phosphorylation, inhibited the activation of JNK and p38, and inhibited the expression of COX-2. In addition, 2% EPA was applied to the naturally aging buttock skin of elderly men thrice a week for 2 weeks, and a skin biopsy was performed 24 h later. The results showed that topical application of EPA to elderly skin increased the procollagen of the entire dermal fibroblasts. Increased expression of tropoelastin and fibrillin-1 and increased expression of TGF-β1, -β2, and -β3 in aged skin proved that EPA has preventive and therapeutic effects on intrinsic skin aging (80).

ASX has investigated the protection effect on UVB-induced oxidative stress and apoptosis in normal human epidermal keratinocytes (NHEKs). NHEKs were pre-treated with ASX for 24 h and then exposed to UVB (280–360 nm) irradiation at UV20–60 mJ/cm^2^. ASX (20 μM) could significantly inhibit UVB-induced NHEK cytotoxicity and ROS production. ROS can activate apoptosis signaling pathways. ASX was demonstrated to effectively reduce the number of early apoptotic cells. The molecular mechanisms could be the inhibition of the expression of apoptosis-related proteins BCL2-Associated X (BAX), Caspase-3 (CASP3), and poly ADP-ribose polymerase (PARP). Results indicated that ASX acted as a ROS scavenger to protect skin from UVB-induced damage (89). ASX is a superior antioxidant, having greater antioxidant capacity than canthaxanthin and β-carotene in human dermal fibroblasts. It was reported that dietary ASX could accumulate in the skin and could reduce UVA-induced wrinkle formation in hairless mice (35). Mice were fed ASX supplements for 70 days, HPLC-PDA analysis showed that ASX could accumulate in both the dermis and epidermis in a dose-dependent manner, and the concentration of ASX in the dermis is ~20–30 times that of the epidermis per square millimeter of skin area. A total of 65 healthy women (aged 35–60 years) were given oral supplements containing 6 mg or 12 mg dose of ASX or a placebo daily for 16 weeks. The study found that wrinkle parameters and skin moisture content significantly worsened in the placebo control group at 16 weeks, while the ASX group maintained the skin level as week 0. The viscoelastic fraction and bioelasticity of the ASX low-dose group were significantly improved at week 16. It shows that long-term supplementation of ASX can preventively inhibit skin deterioration caused by environmental damage, thereby delaying the skin aging process (91). To assess the protective role of astaxanthin for UV-induced skin deterioration, 23 healthy Japanese participants were recruited and assigned to the astaxanthin group supplemented with 4 mg of astaxanthin every day for 9 weeks or the placebo group. The back skin was irradiated with a solar simulator. Compared with the control group, the minimal erythema dose (MED) after ASX supplementation was significantly increased from baseline levels, and the “rough skin improvement” and “texture” of the skin in the non-irradiated areas of the ASX group were also significantly improved compared with baseline (104). These results suggest that KO and its ingredients can be developed as a dietary supplement or for topical use to prevent skin photoaging.

PLs may also have the potential to prevent and treat skin aging. Cell experiments confirmed that PS, lysophosphatidylserine (LPS) and lysophosphatidic acid (LPA) can inhibit the UV-induced decrease in type I procollagen and the increase in MMP-1 in human skin fibroblasts, and PS can block UV-induced IL-6 and COX-2 gene expression dose-dependently (105). The clinical experiment was performed using PS with topical application on human skin *in vivo*, just as Kim's study (80). The young and aged Korean adults were treated with 2% PS on the skin for 24 h. It was found that topical application of PS could prevent the reduction of procollagen and the upregulation of MMP-1 expression in UV-irradiated skin, and can stimulate protein synthesis in young human skin. Notably, PS-treated skin also showed increased procollagen expression in skin without UV exposure. However, PS did not prevent UV-induced expression of TNF-a and IL-1a in young human skin. Results were similar in experiments in the elderly, with PS increasing the expression of type I procollagen and decreasing the expression of MMP-1 in intrinsically aged human skin (105). Clinical trials of dietary supplementation with PUFA found that severe photoaging was inversely associated with a higher intake of ALA in men and with a higher intake of EPA in women. Dietary supplementation and topical use of PUFA have shown good anti-aging effects and can improve both natural aging and UV-induced photoaging (65).

### Maintain skin hydration

Skin moisture is the most important factor in maintaining skin health and controlling aging. The stratum corneum, which affects skin moisturizing, forms a lipid layer composed of ceramides, cholesterol, and free fatty acids between keratinocytes (106). ROS produced by UV exposure causes skin barrier dysfunction, which leads to dry skin. Supplementing with ceramide, free fatty acids, and hyaluronic acid, either applied to the skin or ingested orally, can improve skin hydration.

Ceramide plays a role in maintaining the moisture in the epidermis and skin barrier, and supplementation with dietary sphingomyelin (precursors of ceramides) increases ceramide levels in the body (107). It was demonstrated that oral administration with sphingomyelin-containing milk phospholipid improved skin hydration in a UVB-induced photoaging mice model (106). It was found that milk phospholipids suppressed UV-induced increase in erythema and skin thickness, decreased transepidermal water loss, and increased skin moisture in hairless mice. Milk phospholipids increased the expression of skin moisture-related factors, including filaggrin, involucrin, and aquaporin3 (AQP3). Additionally, hyaluronic acid (HA) content in the skin tissue was increased by regulating the expression of HA synthesis- and degradation-related enzymes. Milk phospholipids suppressed UV-induced decrease in ROS levels and upregulated the expression of the antioxidant enzymes superoxidase dismutase 1 and 2 (SOD1, SOD2), catalase (CAT), and glutathione peroxidase1 (Gpx-1). Moreover, milk phospholipids demonstrated ROS scavenging effects by regulated heme oxygenase-1 (HO-1), an ROS regulator, through activation of nuclear factor erythroid-2-related factor 2 (Nrf2). In summary, sphingomyelin-containing milk phospholipids inhibit loss of skin moisture and reduce damage to skin barriers caused by photoaging by maintaining HA content and reducing ROS levels.

Skin aging often leads to decreased hyaluronic acid, which is a critical component in maintaining skin moisture (99). Fatty acids are essential for skin hydration, and polyunsaturated fatty acid deficiencies increase water loss through the skin barrier (71, 108). Evidence showed that UVB exposure and aging downregulated the expression of genes responsible for hyaluronic acid synthesis (HAS) in the dermis (109). However, oral administration of KO reversed the downregulation of the HAS genes, restored hyaluronic acid content in the skin, prevented skin water loss, and enhanced the moisturizing effect of the skin in UVB-induced skin photoaging mice (4). In HaCaT cells, the moisturizing activity of KO was evaluated by hyaluronic acid synthesis. Compared with the control, KO increased the synthesis of hyaluronic acid, showing a concentration-dependent effect (4). For treating inflammatory skin diseases, such as atopic dermatitis (AD), it was found that feeding AD mice with krill-derived alkyl phospholipids (Alk) for 3–5 weeks significantly reduced the transepidermal water loss (TEWL) of the dorsal skin (37).

The commonality of KO and its ingredients in resisting skin aging is mainly to reduce the production of ROS. Whether these active ingredients can exert synergistic effects in KO and promote anti-aging effects deserves further in-depth research. An experimental study of KO and ingredients on skin anti-aging is summarized in Table 2.

**Table 2:** Experimental study of krill oil and ingredients on skin anti-aging.

**Ingredients**	**Experiments**	**Subjects**	**Manipulations**	**Results**	**Skin benefits**	**References**
KO	Cell experiment	HDF, HaCaT, and B16/F10 cells, with 5 mJ/cm2 UVB irradiation for 2 min	KO 0.25, 0.5, 1, 1.5, 2, 4, and 8 mg/mL for 48h	↑DPPH radical scavenging activity	Anti-skin aging	(4)
KO	Animal experiment	UVB-induced skin photoaging in hairless mice	Oral treatment of KO (100, 200, 400 mg/kg) for 15 weeks	↓Wrinkle length and depth	Anti-skin aging	(4)
n-3 PUFA	Animal experiment	Photoaging mouse model	*n*-3 PUFA/*n*-6 PUFA diet	↑Hmmr (hyaluronic acid receptor) ↓Mmp9	Anti-skin aging	(36)
EPA	Cell experiment	HDFs, Hs27, and HaCaT with UV irradiation	EPA, DHA, AA, LA, and OA for 24 h	↓UV-induced MMP-1 expression	Anti-skin aging	(64)
EPA	Clinical trial	Young Korean adults (20–30 years old) were irradiated with UV light and aged Korean adults (>75 years old)	Topical application of 2% EPA twice (24 h intervals) for young male and 2% EPA for 2 weeks (total of six times) for aged male	↓Epidermal thickening	Anti-skin aging	(80)
EPA	Clinical trial	2,919 subjects aged 45–60 year	Intake of ALA, EPA, DPA, and DHA were evaluated by dietary source	Inverse associations between severity of photoaging and ALA intake in men, and between severity of photoaging and EPA intake in women.	Anti-skin aging	(65)
				No significant association was found between photodamage and DPA or DHA.		
ASX	Cell experiment	NHEKs exposed to UVB irradiation	ASX (20 μM) for 24 hours	↓UVB-induced NHEKs cytotoxicity	Anti-skin aging	(89)
ASX	Animal experiment	Female hairless mice exposed to UVA	Diet with 0.01% or 0.1% ASX monoester	↓Transepidermal water loss (TEWL)	Anti-skin aging	(35)
ASX	Cell experiment	Keratinocytes and fibroblasts irradiation with UVB	Treated with 0, 1, 5, or 10 μM ASX for 4 h	↓IL-1α, IL-6, IL-8 and TNF-α levels	Anti-skin aging	(91)
ASX	Clinic trial	Participants aged from 30 to 60 years irradiated with UVB	Take 4 mg of ASX for 9 weeks	↑MED	Anti-skin aging	(104)
PLs	Animal experiment	UVB-induced photoaging hairless mice	Orally administered with milk phospholipids (50, 100, and 150 mg/kg) once a day for 8 weeks	↓UV-induced erythema and skin thickness	Anti-skin aging	(106)
PS	Cell experiment	Hs27 with UV irradiation	Treat with LPS, LPA, LPG,	↑Type I procollagen	Anti-skin aging	(30)
				↓IL-6, COX-2		

## Krill oil on alleviating skin inflammation

Inflammation is a protective physiological response to internal or external damage to the body. The skin is a protective organ, and the cutaneous immune system responds to inflammatory stimuli. The skin immune system includes innate immunity and adaptive immunity. The skin contains a variety of innate immune cells and mediators as well as phagocytes, such as antimicrobial peptides, proteins of the complement system, neutrophils, and macrophages. Pathological inflammatory skin diseases may develop into chronic inflammation when the skin's adaptive immune system is dysfunctional. KO has demonstrated the ability to relieve inflammation-related skin disorders, including UV-induced skin inflammation, atopic dermatitis, and psoriasis. This beneficial effect is attributed to its ability to strengthen the stratum corneum lipid barrier and maintain the correct intercellular lipid balance (Figure 2).

KO has been proven to have anti-inflammatory properties. A combined dietary supplement containing multiple natural antioxidants and/or anti-inflammatory drugs, including KO, coenzyme Q10, lipoic acid, resveratrol, grape seed oil, α-tocopherol, and selenium, was investigated *in vitro* study on the prevention of skin disorders by Fasano et al. Each ingredient was administered to human keratinocytes alone or in combination. It was found that these compounds, including KO, whether used in combination or alone, including KO, can produce anti-inflammatory effects on HaCaT keratinocytes treated with TNF-α. The compound modulates the nuclear factor-κB (NF-κB) pathway, which is closely related to pro-inflammatory cytokine synthesis, and significantly reduces the production of IL-6 and monocyte chemoattractant protein-1 (MCP-1)—one of the most expressed cytokines by keratinocytes during inflammation. This study further demonstrates the potential of KO to be applied in topical formulations as it can overcome issues related to oral formulations, which is biodistribution and metabolic transformation, and directly target keratinocytes (110).

### UV-induced skin inflammation

UV radiation can cause skin inflammation. When human skin is acutely exposed to sunlight, UV radiation results in a sunburn reaction characterized by erythema, edema, itching and pain. Histopathological changes include thickening of the stratum corneum and the formation of apoptotic epidermal keratinocytes. Changes in immune cells include dermal neutrophil, lymphocyte, and monocyte infiltration, leading to changes in inflammatory cytokines, such as increasing the amount of tumor necrosis factor-α (TNF-α) and the pro-inflammatory cytokine IL-1 in the entire human skin tissue, and decreasing the expression of the anti-inflammatory cytokine IL-10 (9, 111). This is mediated, in part, by lipids such as pro-inflammatory eicosanoids. Lipids actively participate in or mediate multifaceted effects on skin and immune cells residence and infiltration in inflammatory settings.

When UV rays irradiate the skin, polymorphic neutrophils (PMNs) and neutrophils are recruited into the damaged tissue. Myeloperoxidase (MPO), a cytotoxic enzyme that activates inflammation, is related to both PMNs and neutrophils and can be released by PMNs (112–115). Studies have found that oral administration of KO can reduce UV-induced skin inflammation. Evaluate the effect of KO on skin edema by measuring the weight of 6 mm diameter skin samples. It was found that the average skin weight of mice in the KO group was significantly decreased in a dose-dependent manner. Moreover, KO directly inhibits MPO activity, resulting in a reduction in the number of neutrophils recruited to the inflammatory site. KO increased the expression of anti-inflammatory cytokine IL-10 and decreased pro-inflammatory cytokine IL-1β. Therefore, KO treatments balance pro- and anti-inflammatory cytokines and modulate the inflammatory response induced by UVB radiation effects (4).

Clinical studies have shown that intake of *n*-3 PUFA can reduce the pro-inflammatory and immunosuppressive prostaglandin E2 (PGE2) production in human skin exposed to UV radiation, reduce erythema development, and protect the skin from immunosuppression. It was supposed that supplementation ω- 3 fatty acids could reduce the occurrence of non-melanoma skin cancer in high-risk populations (116). In clinical trials, ASX plays a photoprotective role by inhibiting UVB-induced skin inflammation (117). These anti-inflammatory or anti-cancer active ingredients are main components of KO, and their beneficial mechanisms of skin health can be used as a reference for further research on KO's skin protection.

### Psoriasis

Psoriasis is also a chronic inflammatory skin disease that generally manifests as skin thickening, scales, and erythema. It can occur in various parts of the body. Currently, there is no complete cure. It can only be managed through the use of topical or systemic drugs, which affect the patient's life quality.

Alterations in fatty acid composition are observed in psoriasis, and dietary supplementation may have the potential to improve fatty acid metabolism (11). Vijayapoopathi et al. evaluated the antipsoriasis effects of KO in combination with cannabidiol and myo-inositol using a mouse model of IMQ-induced psoriasis. Mice were orally treated with nutraceutical combination twice a day for 7 consecutive days. The clinical psoriasis area and severity index (PASI) was used to score the severity of skin inflammation on the back of mice. The study found that the treatment delayed the onset of erythema and skin scaling when compared with the model group. Mice treated with the KO combination displayed reduced scales, erythema, skin thickness, ear thickness, and vascularity, along with mild-to-moderate PASI scores, indicating that KO combined with myo-inositol and cannabidiol can significantly improve IMQ-induced psoriasis in mice (118). In Skroza et al.'s research, 40 patients with psoriasis and metabolic syndrome were treated with nutritional supplements containing coenzyme Q10 and KO. Results showed that nutraceuticals restored both psoriasis and normal lipid profile (119).

### Atopic dermatitis (AD)

Atopic dermatitis (AD) is an inflammatory skin lesion accompanied by symptoms such as itching, skin buckling, erythema, papules, skin vesicles, and skin thickening. AD is a type of allergy caused by the dysfunction of the barrier function of the skin. It affects more than 15% of children in developed countries. AD is associated with various factors, including immunological abnormalities and exposure to allergens.

The occurrence of atopic dermatitis is associated with reduced *n*-3 PUFA intake and increased *n*-6 PUFA intake. Koch et al. conducted a randomized double-blind controlled trial to explore whether *n*-3 PUFA can improve atopic dermatitis. The experimental group was supplemented with 5.4 grams of DHA per day, and the control group was supplemented with saturated fatty acids of the same energy. The results found that DHA significantly improved the SCORAD (Severity Score of Atopic Dermatitis) index. After intervention treatment, although the activation status of peripheral blood mononuclear cells (PBMCs) was adjusted in both groups, anti-CD40/interleukin 4-mediated IgE synthesis by PBMCs was significantly reduced only in the DHA group. In addition, among plasma fatty acids, *n*-3 PUFA increased and *n*-6/*n*-3 PUFA ratio decreased in the DHA group (120). Oral EPA and DHA can also promote wound healing by improving chronic inflammation. Chronic venous lower extremity ulcers (CVLUs) patients consumed 2.5 grams of EPA and 0.5 grams of DHA every day. After 56 days, the levels of matrix metalloproteinase-8 and human neutrophil elastase in the CVLU fluid of the treatment group were significantly reduced, as well as the proportion of CD66b+ cells and the wound area was significantly reduced. The reduction was significantly inversely correlated with CD15+ cells and CD66b+ cells in wound fluid. These cytokines, associated with activated polymorphonuclear leukocytes (PMNs) and their derived proteases, destroy newly developing tissue and degrade key growth factors. This study confirmed that oral treatment with EPA and DHA can improve chronic inflammation and promote the healing of CVLU wounds by regulating PMN activity (121).

Oxidative stress has been reported to play an important role in the pathophysiology and exacerbation of AD symptoms, and the use of antioxidants has been shown to be beneficial in protecting against the harmful effects of increased oxidative stress. ASX has strong antioxidant activity and anti-inflammatory effects to improve dermatitis. NC/Nga mice were used to induce an AD model by mite treatment. ASX (100 mg/kg) was administered orally once daily and thrice weekly for 26 days. Results showed that ASX significantly reduced clinical skin severity scores and reduced spontaneous scratching in AD mice. Skin histological analysis showed that the number of eosinophils, total mast cells, and degranulated mast cells in the skin of mice was significantly reduced by ASX treatment. Serum IgE levels were significantly reduced, and the mRNA and protein levels of eotaxin, migration inhibitory factor (MIF), IL-4, IL-5, and L-histidine decarboxylase were significantly reduced in ASX-treated mice. These results indicated that ASX improved dermatitis and pruritus by regulating inflammation and the expression of inflammatory cytokines (122). ASX is one of the main components of KO. The mechanism of ASX improving dermatitis may be one of the pathways through which KO works.

With the occurrence of AD, the total amount of phospholipids and the proportion of plasmalogen (PLs) in plasma decreased. PLs are an endogenous defense factor that protects the skin from oxidative stress and are in a high concentration in the skin. Krill contains a relatively high concentration of alkyl phospholipids (Alk), which is the precursor of PLs. Watanabe et al. supplemented AD-infected NC/Nga mice with phospholipid-enriched Alk extracted from krill for 3–5 weeks and found that supplementation of Alk can strengthen the skin intercellular lipid barrier and alleviate AD. After feeding Alk to AD mice, the contents of 20:5 (EPA) and 22:6 (DHA) at sn-2 position of PLs in serum were increased, while 20:4 (arachidonic acid) was decreased at this position. This showed that Alk alleviated inflammation by reducing arachidonic acid binding to PLs and increasing DHA and EPA binding to PLs (37). Ceramide is the main lipid component of the lamellar sheets present in the intercellular spaces of the stratum corneum and plays an important role in building and maintaining the skin's water-permeable barrier function (123). Pro-inflammatory cytokines were supposed to alter the activity of ceramide-metabolizing enzymes, thereby changing the ceramide composition of the skin and further leading to skin barrier damage (124). The composition of ceramides in the skin is changed in AD, which can be reversed by Alk. It was found that the ratio of non-hydroxy ceramides and hydroxy ceramides in AD mice treated with Alk was similar to that in normal mice, while it was not observed in AD mice treated with fish oil (37). The experimental study of KO and ingredients for alleviating skin inflammation is summarized in Table 3.

**Table 3:** Experimental study of krill oil and ingredients on alleviating skin inflammation.

**Ingredients**	**Experiments**	**Subjects**	**Manipulations**	**Results**	**Skin benefits**	**References**
KO	Cell experiment	HaCaT human immortalized keratinocytes, NCTC 2544 human immortalized keratinocytes	A combination (coenzyme Q10, KO, lipoic acid, resveratrol, grape seed oil, α- tocopherol, and selenium), each component was administered, alone or in combination, KO (0.8, 4.0, and 20.0 μg/mL)	ROS	Anti-inflammation	(110)
KO	Animal experiment	UVB-induced skin photoaging in hairless mice	Oral treatment of KO (100, 200, 400 mg/kg) for 15 weeks	↓Wrinkle length and depth	Anti-skin aging	(4)
KO	Animal experiment	The imiquimod (IMQ)-induced psoriatic mice model	Oral treatment with EPA 1000 mg, EPA 500 mg, or KO 1000 mg twice daily for 7 days	↓The Psoriasis Area Severity Index (PASI) score	Anti-inflammation	(118)
DHA	Animal experiment	Female hairless mice model with UVB-induced dermal papilloma formation skin	Applied topically with 200 μL of acetone or DHA (10 μmol) to the dorsal skin 30 min	↓UVB-induced expression of P-STAT3 (Tyr705)	Anti-inflammation	(72)
DHA	Clinical trial	53 patients suffering from atopic eczema aged 18–40 years	Oral treatment with DHA 5.4 g daily for 8 weeks	↓Anti-CD40 /interleukin 4-mediated IgE synthesis of PBMC	Anti-inflammation	(120)
EPA+DHA	Clinical trial	40 participants between 18 and 81 years of age have at least one existing CVLU between the ankle and knee for ≥3 months	Oral treatment with 2.5 g EPA and 0.5 g DHA each day	↓Percentage of CD66b+ cells	Anti-inflammation	(121)
ASX	Animal experiment	The mite-induced AD NC/Nga mice model	Oral treatment with 100 mg/kg ASX 3 times a week for 26 days	↓The clinical skin severity score	Anti-inflammation	(122)
PLs	Animal experiment	Atopic dermatitis(AD) induced by *Myobia musculi* infection of NC/Nga mice model	28.8% alk diet for 3 and 5 weeks	↓Transepidermal water loss (TEWL)	Anti-inflammation	(37)

## Krill oil on wound healing enhancement

As a barrier organ that protects the body, the skin has a complex self-repair process, which can quickly and effectively promote wound healing. Wound healing is divided into four phases, including hemostasis (blood clot formation), inflammation (infiltration of immune cells), proliferation (angiogenesis, granulation, epithelialization, and ECM remodeling through proliferation and migration of fibroblasts and keratinocytes), as well as skin layer maturation (wound contraction and inflammation resolution). The initial inflammatory phase is characterized by platelet activation, the recruitment of immune cells, the release of growth factors and cytokine, followed by the secretion of growth factors and enhanced cell proliferation, and finally, in a remodeling phase, collagen production and histogenesis leads to mature scars (125). While the skin is susceptible to a variety of internal and external factors, when the skin is irritated by exposure to the environment, it increases inflammation, resulting in ulcerative chronic wounds, hypertrophic scars, or keloids. Cracked and dry skin is often accompanied by changes in skin lipids and loss of moisture. Physiological factors such as stress influence the wound healing process by affecting blood flow, skin metabolic and immune status, as well as hair follicle function through neurohormonal and steroid hormone levels. KO has been shown to promote wound healing by improving the function of fibroblasts and repairing endothelial angiogenesis injury. *n*-3 fatty acids and other active ingredients in KO help reduce inflammation, and promote cell proliferation and angiogenesis, thereby accelerating the wound healing process (Figure 2).

Some cases such as diabetes and aging can damage the wound-healing process and result in chronic, non-healing wounds (126). Diabetes mellitus (DM) can lead to excessive and prolonged inflammation, which, in turn, impairs wound repair and affects wound healing. At present, there are still few effective methods to treat diabetic wound healing (127), but KO has been shown to improve skin conditions and promote wound healing. Studies have found that topical application of KO could significantly accelerate wound healing in type 2 diabetes mellitus mice by reversing the symptoms of excessive inflammation, impaired collagen deposition, and inhibited neovascularization in diabetic mice (128). Diabetic mice induced by streptozotocin and a high-fat diet received dorsal skin incision to establish a diabetic wound model. It was found that KO treatment reduced the width and area of the wound, and promoted skin thickening and hair follicle formation at the wound (128). Furthermore, KO increased CD31, a marker of endothelial cells (ECs), and also significantly activated the expression of *Angpt2, Angpt4, Vegf-a*, and *E-Cadherin* in the skin, indicating that KO enhanced neovascularization in diabetic wounds (128). Moreover, collagen deposition is important for wound healing. TGF-β1 and α-SMA are sensitive factors for fibroblast differentiation, and their expression is inhibited under DM, thereby affecting collagen formation. KO can reactivate the expression of Tgf-β1 and Acta2 in the skin and accelerate the accumulation of collagen in diabetic wounds (128). Other studies have found that oral administration of KO significantly promoted wound healing of colonic anastomosis after colectomy in rats under the influence of inflammatory cell infiltration, fibroblast activity, angiogenesis, and collagen deposition (129).

Fatty acids are key components for cell membrane structure and function and play an active role in skin health. Peng et al. used mixed oil SMOF (soybean oil, medium-chain triglyceride, olive oil, fish oil), which was rich in *n*-3 PUFA, for wound healing in rats. SMOF was administered intravenously to rats with wound excision on the back. It was found that SMOF decreased the wound area significantly from 72 h. The level of IL-10 in the SMOF group increased after 48 h of treatment. And the distribution rate of fibroblasts and collagen fibers in the SMOF group is higher than the control group. It shows that SMOF rich in *n*-3 PUFA can promote wound healing. And this may be related to the anti-inflammatory effect of *n*-3 PUFA (130). In addition, local treatment with DHA combined with pigment epithelium-derived factor (PEDF) can selectively recruit type 2 macrophages and prevent neutrophil infiltration from diabetic corneal injury, thereby promoting corneal nerve regeneration and wound healing in diabetic mice (131). EPA can promote neovascularization mediated by endothelial progenitor cell migration (132).

ROS are involved in all phases of wound healing. ASX, a powerful antioxidant, has proved to be an effective compound for accelerating wound healing. In lipopolysaccharide-stimulated fibroblasts, ASX eliminated ROS and inflammatory effects and improved the cell viability and proliferative capacity (133). It was reported that ASX could enhance gingival wound healing after hyperglycemia-induced oxidative stress through its antioxidant properties (134). Healthy female mice who received full-thickness dermal wounds were topically treated with ASX twice daily for 15 days. ASX-treated wounds showed contraction by day 3 and complete wound closure by day 9. The expression of wound healing biological markers such as collagen type I alpha 1 (Col1A1) and basic fibroblast growth factor (bFGF) was increased, but the expression of oxidative stress marker inducible nitric oxide synthase (iNOS) was decreased in ASX-treated mice (135).

It has been reported that proteolytic enzymes can cause the debridement of wounds and the removal of necrotic tissue. A multienzyme preparation isolated from Antarctic krill has intense proteolytic activity on protein substrates. It is more active than other commonly used proteolytic agents for wound debridement and has sound effects on ulcerative wounds. The effect of debridement was confirmed by cell experiments, animal experiments, and clinical experiment (136–139). Overall, KO may control excessive inflammation and oxidative stress in the skin, promote new blood vessels and collagen production, and promote wound healing. The experimental study of KO and ingredients on wound healing enhancement is summarized in Table 4.

**Table 4:** Experimental study of krill oil and ingredients on wound healing enhancement.

**Ingredients**	**Experiments**	**Subjects**	**Manipulations**	**Results**	**Skin benefits**	**References**
KO	Animal experiment	Two perforated wounds on the upper back and lower back, respectively, in streptozotocin (STZ) and high-fat diet (HFD)-induced type 2 diabetes mice	KO ointment with a concentration at 0.3 mg/g for 16 days	↓The wound width and area	Promote wound Healing	(128)
KO	Animal experiment	Colon anastomoses rat model	Oral treatment with 100mg/kg KO capsule (KO 500 mg + EPA 66 mg + DHA 35 mg + coline 25 mg + ASX 40 mg) for 3 and 7 days	↑Bursting pressure and hydroxyproline measurements	Promote wound Healing	(129)
Omega-3 PUFA	Animal experiment	An excisional wound was created on the dorsum skin of SD rats aged 13 weeks and weighed 200–250g	Administered intravenously 20% SMOF lipid, 30% comprising soybean oil, 30% MCTs, 25% olive oil and 15% fish oil, 0.2 mL/kg bw/h for 72h	↓The wound area	Promote wound Healing	(130)
DHA	Animal experiment	The corneal injury caused by the removal of the central epithelium and anterior stroma of the cornea in diabetic mice	Topically application with PEDF (0.4 ng) plus DHA (80 ng) 3 times per day for 2 weeks	↑The density of SP-positive nerves	Promote wound Healing	(131)
EPA+DHA	Clinical trial	40 participants between 18 and 81 years of age have at least one existing CVLU between	Oral treatment with 2.5 g EPA and 0.5 g DHA each day	↓Percentage of CD66b+ cells	Anti-inflammation	(121)
				↑Healing of CVLUs		
ASX	Cell experiment	Human gingival fibroblasts exposed to high glucose	100 μM ASX for 24h	↓The ROS levels	Promote wound Healing	(134)
ASX	Animal experiment	Full-thickness dermal wounds were created in the healthy female mice	Topically application with 78.9 μM ASX extraction twice daily for 15 days	↑The wound area	Promote wound Healing	(135)
PLs	Animal experiment	AD induced by *Myobia musculi* infection of NC/Nga mice model	28.8% alk diet for 3 and 5 weeks	↓Transepidermal water loss (TEWL)	Anti-inflammation	(37)

## Conclusions

**Figure 3:**
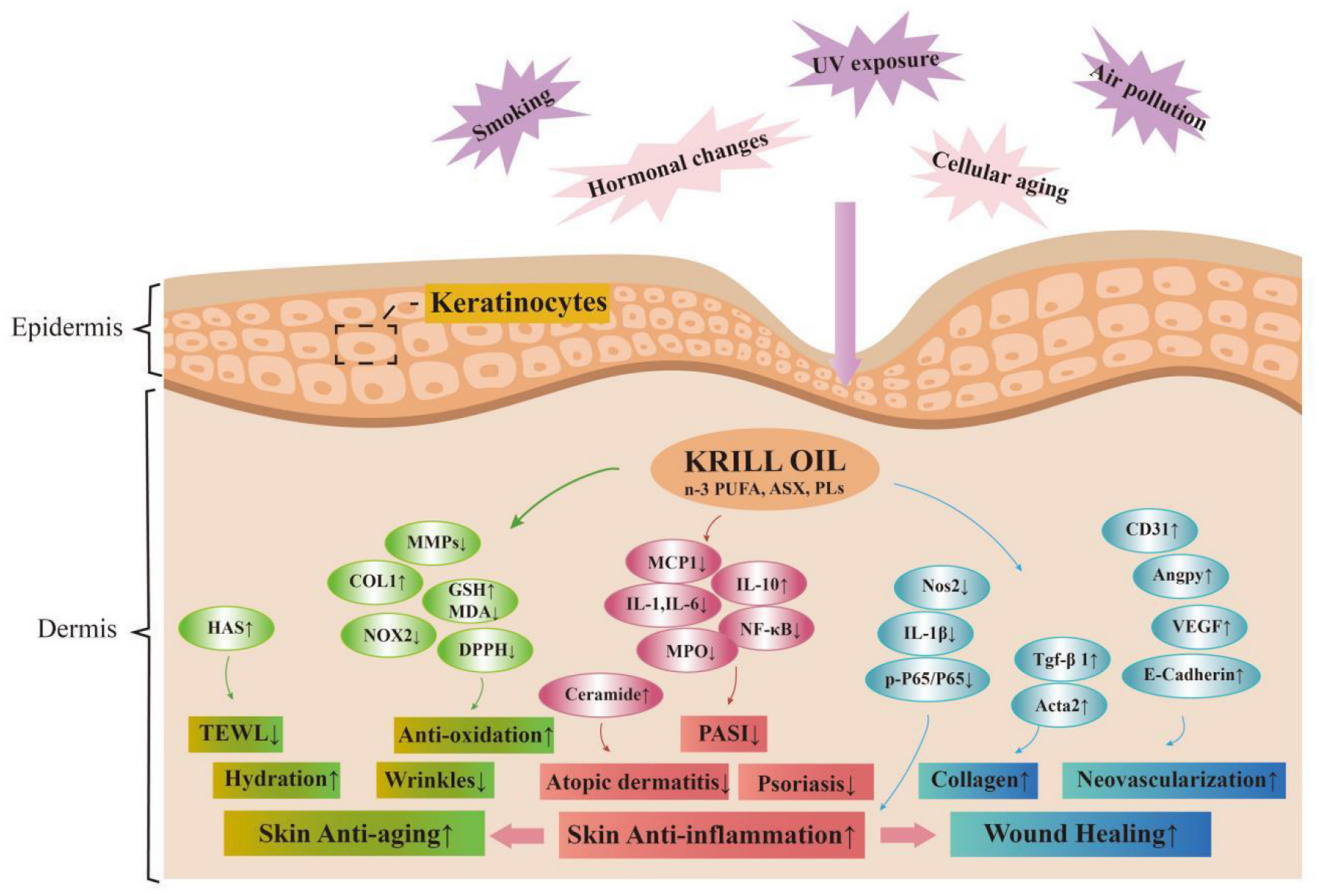
Mechanisms of krill oil's role on skin health and problems. Skin aging is categorized into two types: (1) intrinsic aging, which is influenced by hormonal changes and cellular aging, and (2) extrinsic aging, which results from external factors like UV exposure, air pollution, and smoking. KO and its functional ingredients decelerate skin aging processes through antioxidation, anti-inflammation, thereby protecting skin barrier, fostering wound healing, maintaining hydration, and promoting youthful and healthy skin. KO, krill oil; *n*-3 PUFA*, n*-3 polyunsaturated fatty acids; DHA, docosahexaenoic acid; EPA, eicosapentaenoic acid; ASX, astaxanthin; PLs, phospholipids; HAS, hyaluronic acid synthesis; MMP, matrix metalloproteinases; COL1, type I collagen; GSH, glutathione; MDA, malondialdehyde; DPPH, 2,2-diphenyl-1-picrylhydrazyl; MCP-1, monocyte chemoattractant protein-1; IL-1, interleukin-1; IL-6, interleukin-6; IL-10, interleukin-10; MPO, myeloperoxidase; NF-κB, nuclear factor kappa-B; Nos2, nitric oxide synthase 2; IL-1β, interleukin-1beta; P65, NF-κB p65 protein; p-P65, phospho-NF-κB p65 protein; Tgf-β1, transforming growth factor beta 1; Acta2, actin alpha 2; CD31, platelet endothelial cell adhesion molecule-1; Angpy, angiopoietin; VEGF, vascular endothelial growth factor; TEWL, trans-epidermal water loss; PASI, psoriasis area severity index. ↑, stands for enhancement or activation; ↓, stands for weakening or inhibiting.

Increasing studies support the beneficial effects of KO on skin health, including anti-aging, anti-inflammatory properties, and wound healing promotion, which have been preliminarily evaluated in clinical, animal, and cellular experiments (Tables 2–4). The potential mechanisms of action are summarized in Figure 3. This indicates that KO has great potential as a skin protectant. However, further research is needed to understand the specific effects of KO on the skin under environmental stress and its potential to improve skin condition, as well as to elucidate the molecular mechanisms underlying the multiple benefits of KO on skin health. In addition, the improvement of technological processes for green extraction and transportation systems will support consumer's growing interest and demand for efficacious, safe, natural, and sustainable natural alternative skincare solutions (140). In summary, the application of KO as a therapeutic measure represents an innovative and efficient approach that can be incorporated into future skin health treatments and improvements. Further illumination of its biological function and the underlying mechanisms, and translation of the basic research findings into novel skin health interventions, dietary supplements, and therapeutic approaches for preventing chronic diseases will certainly be the focus of research for years to come.

## Author contributions

LD: Writing – original draft, Visualization, Investigation. JY: Writing – review & editing, Project administration, Conceptualization. XW: Writing – original draft, Visualization. GZ: Writing – original draft, Visualization. JZ: Writing – review & editing, Project administration, Conceptualization. HZ: Writing – review & editing, Writing – original draft, Visualization, Methodology, Conceptualization. ZW: Writing – review & editing, Supervision, Conceptualization. YL: Funding acquisition, Writing – review & editing, Supervision, Conceptualization.
